# Supplementary material for: The causal relationship between the human gut microbiota and pyogenic arthritis: a Mendelian randomization study
Source: Front Cell Infect Microbiol. 2024 Nov 26;14:1452480. doi: 10.3389/fcimb.2024.1452480 (PMC11629706; doi:10.3389/fcimb.2024.1452480)
Supplement: Supplementary file 1 [file DataSheet1.zip › mendelian test/ebi-a-GCST90027571.csv_forest.pdf]

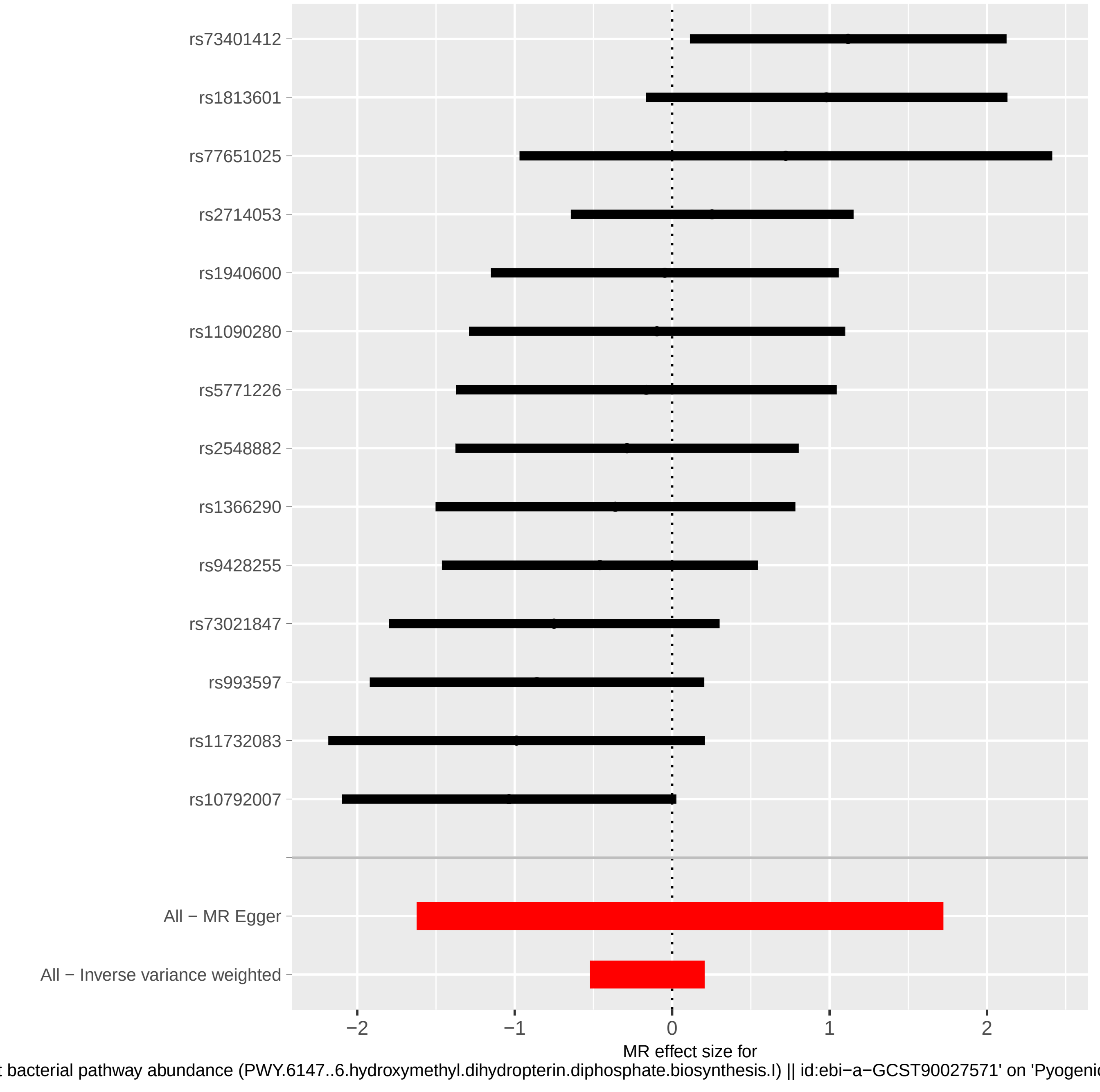
rs73401412
rs1813601
rs77651025
rs2714053
rs1940600
rs11090280
rs5771226
rs2548882
rs1366290
rs9428255
rs73021847
rs993597
rs11732083
rs10792007
All − MR Egger
All − Inverse variance weighted
−2
−1
0
1
2
MR effect size for
t bacterial pathway abundance (PWY.6147..6.hydroxymethyl.dihydropterin.diphosphate.biosynthesis.I) || id:ebi−a−GCST90027571' on 'Pyogenic
